# Method for generating high-quality tetrahedral meshes of geological models by utilizing CGAL

**DOI:** 10.1016/j.mex.2020.101061

**Published:** 2020-09-10

**Authors:** Bowen Wang, Gang Mei, Nengxiong Xu

**Affiliations:** School of Engineering and Technology, China University of Geosciences (Beijing), Beijing, China

**Keywords:** Computational geometry, Geological model, Mesh generation, Tetrahedral mesh, Algorithm, CGAL

## Abstract

High-quality computational meshes are crucial in the analysis of displacements and stabilities of rock and soil masses. In this paper, we present a method for generating high-quality tetrahedral meshes of geological models to be used in stability analyses of rock and soil masses. The method is implemented by utilizing the Computational Geometry Algorithms Library (CGAL). The input is a geological model consisting of triangulated surfaces, and the output is a high-quality tetrahedral mesh of the geological model. To demonstrate the effectiveness of the presented method, we apply it to generate a series of computational meshes of geological model, and we then analyse the stabilities of the rock and soil slopes on the basis of the generated tetrahedral mesh models. The applications demonstrate the effectiveness and practicability of the present method.

• A method for generating high-quality tetrahedral meshes of geological models is presented.
• We evaluate the quality of the tetrahedral mesh of geological model using four metrics.
• Three applications demonstrate the effectiveness and practicability of the presented method.

## Graphical abstract

**Figure:**
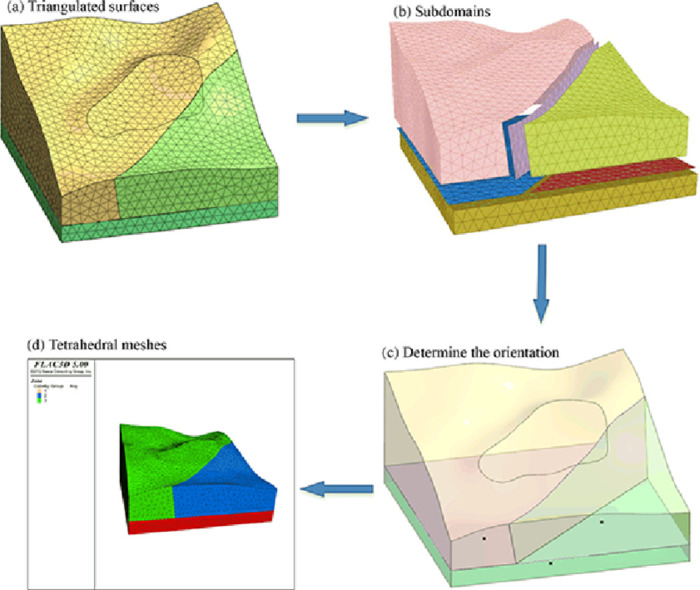

Specifications table

**Table:** 

**Subject Area**	Geotechnical Engineering
**More specific subject area**	Mesh Generation and Numerical Simulation
**Method name**	GeoTet
**Name and reference of original method**	**N/A**
**Resource availability**	N/A

## Method details

In this section, we will describe the implementation details of presented method for generating high-quality tetrahedral meshes of geological models.

## Presented method for generating high-quality tetrahedral meshes of geological models

**Fig. 1:**
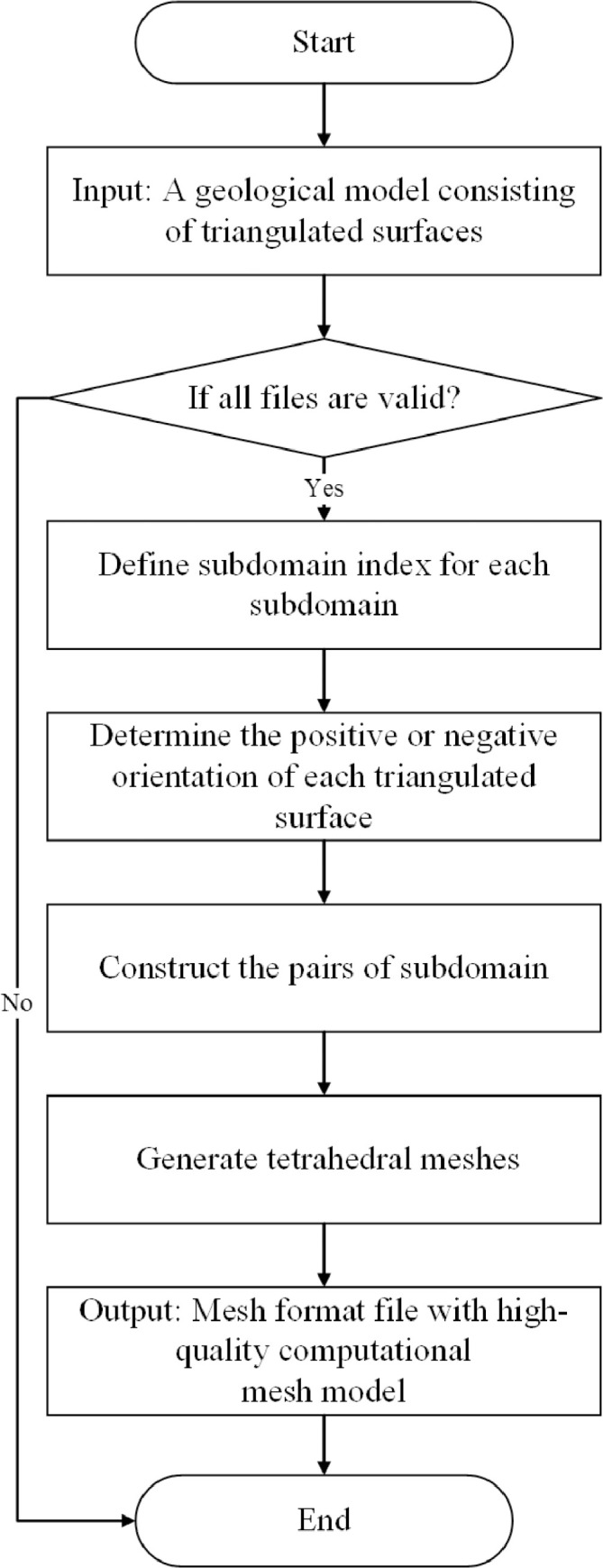
Workflow of the presented method.

The presented method is devoted to tetrahedral mesh generation of a geological model, see Fig. 1. The domain representing the geological model is typically composed of several strata, each one must be the union of at least one connected component of the complement of the 2D triangulated surface. Subdomain indices belonging to the corresponding subdomain must be defined and the orientation of each surface patch should be determined. Before the meshing process, the input pairs of the subdomain should be constructed according to the orientation and the subdomain indices, which represent the subdomains on both sides of the triangulated surfaces.

**Fig. 2:**
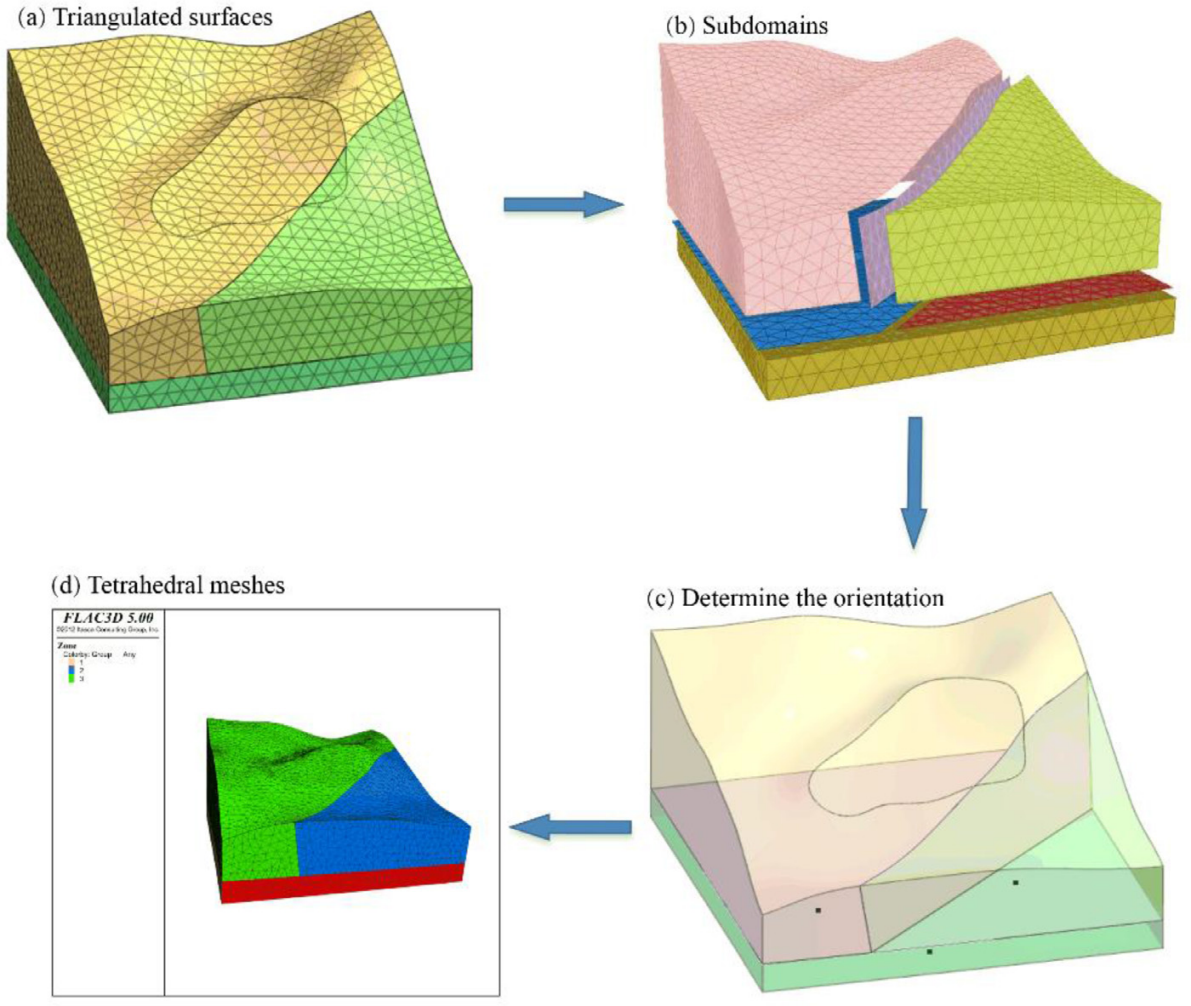
Illustration of the process of generating the tetrahedral mesh of a geological model. (a) All the surfaces must be triangulated and guaranteed the geometric and topological consistency. (b) The domain is divided several subdomain, each subdomain is identified by a subdomain index. (c) Each triangulated surfaces is orientated. We utilize any point located in subdomain to determine the positive or negative orientation. (d) The geological model is meshed with tetrahedrons, which can be used as the computational model in numerical analysis.

The meshing process will preserve the features of the geological model. The mesh generator is customized to output the meshes that cater to the user's needs as much as possible, such as the mesh size and user-customized quality criteria in Fig. 2. The output meshes are composed of tetrahedral meshes, which approximate each input domain feature: subdomains, boundary triangulated surfaces or input domain features with dimensions of 0 or 1.

### Step 1: Inputting a geological model consisting of triangulated surfaces

**Fig. 3:**
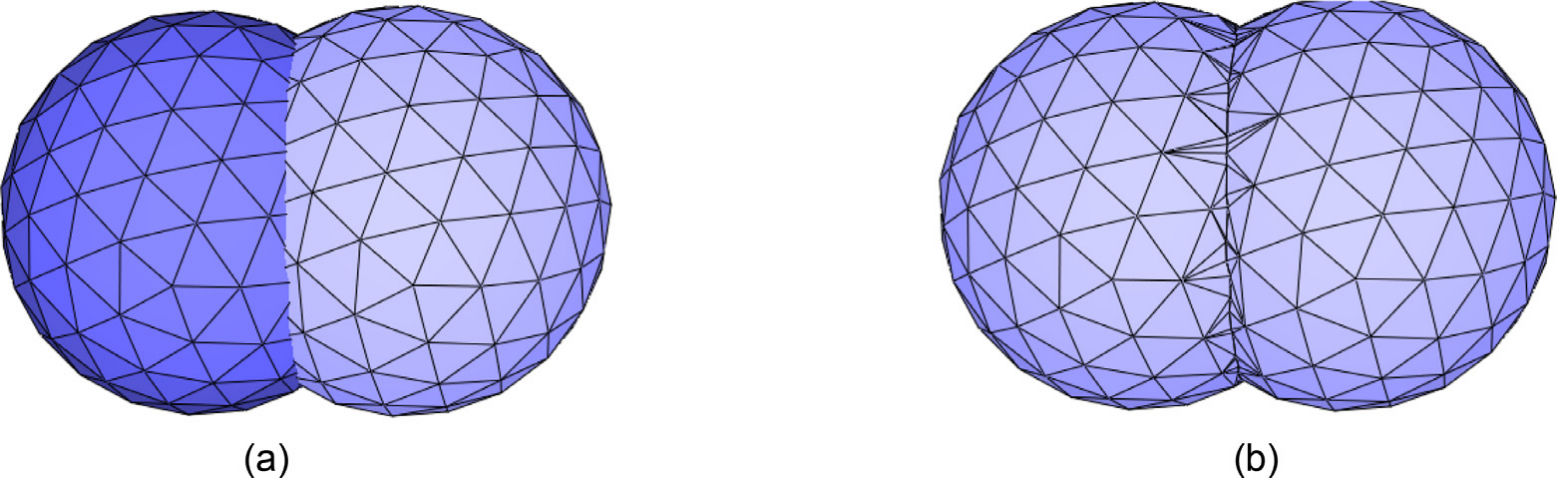
(a) The two triangulated surfaces are not valid inputs since the topological consistency is not promised. (b) The two triangulated surfaces are valid inputs. Geometric and topological consistency are guaranteed.

The geological model to be meshed is enclosed by a collection of triangulated surfaces, forming a complex as in Fig. 2(a). Each triangle of the triangulated surface is called a facet. There are two constraints on triangulated surfaces. First, triangulated surfaces of the complex cannot self-intersect. Second, two triangulated surfaces of the complex are either disjoint or share a subset of their border edges. Fig. 3(a) shows invalid input. The intersection line cannot guarantee topological consistency. There are many isolated points. In this method, isolated points are forbidden. Fig. 3(b) shows valid input. Thus, an important precondition for the meshing algorithm to work correctly is that any two surface patches cannot intersect. If two surfaces intersect, the intersection edges must be part of the input surfaces and have 1-dimensional features.

### Step 2: Determining the orientation of each triangulated surface

The orientation of each triangulated surface must be identified. Each triangulated surface is oriented and has two sides. Each triangulated surface has a positive orientation and a negative orientation. The orientation of each surface is the same as that of all its facets. The positive side is the union of the positive side of each triangulated facet, usually called the "exterior" of the surface. The negative side is the other side of the surface. We can utilize any point located in the subdomain to determine the orientation of the triangulated surfaces in Fig. 2(c).

The function CGAL::orientation (p, q, r, s) in CGAL can be used to determine the orientation of a plane composed of the point p, q, r, which is composed of a triangle in the surface patch. Point s is located on the left side or right side of this triangle. Therefore, taking the three points of facets of triangulated surfaces and a point located in the subdomain as the function argument, we can determine the orientation.

### Step 3: Constructing the input pairs of subdomains

In a geological model, each subdomain represents the corresponding stratum. The subdomains have indices of integer type, and the exterior of the mesh domain is associated with the subdomain index 0. Each subdomain index ranging from 1 to the number of subdomains should be defined. It is also important that for each triangulated surface, the subdomain indices on both sides of the surface are known. The input pairs of subdomains are used to combine the subdomain indices of both sides of each triangulated surface. One subdomain index is on the positively oriented "side" of the triangulated surface. The other subdomain index is on the negative side. The interface of sub-domains is supposed to be provided and sub-domains are sharing input interfaces with different subdomain indices. Besides, we also mark corresponding subdomain indices for the interface. For each triangulated surface, there is a corresponding input pair of subdomain indices. The order of the constructed input pair of triangulated surfaces should be consistent with the order in which the triangulated surfaces are saved. These input pairs of subdomain indices associating the subdomain with triangulated surfaces can guarantee correct mesh generation for each subdomain.

### Step 4: Generating tetrahedral meshes

To achieve the user's needs with respect to the size of the mesh elements and the accuracy of the boundary approximation, we applied some criteria implemented by CGAL. The criteria can drive the Delaunay refinement process and mainly contain the cell size and facet size. The cell size controls the size of the tetrahedral mesh and the facet size controls the size of the surface facets. The mesh generation process is a Delaunay refinement process followed by an optimization. The optimization phase contains four optimization processes: Optimal Delaunay Triangulation (ODT) smoothing [1], a Lloyd smoothing [2], a perturber and an exuder [3]. It should be executed as follows: an ODT smoother, a Lloyd smoother, a sliver perturber, and a sliver exuder. ODT is optimal Delaunay triangulation. The process of each application can be activated based on the needs of the user. After the optimization phase, the average mesh quality will be improved. When the program finishes, it will generate high-quality tetrahedral meshes, such as that in in Fig. 2(d), and will output a mesh format file containing the point, triangle and tetrahedral mesh information.

In general, the surface mesh is typically fixed during the volume mesh generation. When the required volume size is less than half the size of the surface meshes, the method will refine the surface meshes for the generation of the required volume sizes by the package. When the required volume size is greater than half the size of the surface meshes and less than the size of surface meshes, the original surface meshes are fixed, and the refinement is ignored. And the output will be high-quality mesh. Thus, we recommend that the size of the required volume and the surface meshes are as consistent as possible. When the required volume size is greater than the size of the surface meshes, the method will generate ill-shape meshes which can influence the result of numerical simulation. Thus, we recommend that the required volume size should less than or equal to surface meshes size.

The conformity of surface mesh means that there is no isolated point. The method will automatically guarantee the conformity in the refinement process so that the user does not need to do additional settings. Only if the initial surface meshes is conformal, the method will generate meshes with overall conformity.

## Method validation

### Applications of the Presented Method

To demonstrate the effectiveness of the presented method, we apply it to generate a series of computational meshes of geological model. FLAC^3D^ [4] is often used for numerical simulation in geotechnical engineering applications. We also developed an application interface to convert the mesh format file generated by CGAL to a FLAC^3D^ format file. We then analysed the stabilities of the rock and soil slopes on the basis of the generated tetrahedral mesh models.

The measurement metrics of the element quality include (1) the qualitytest1, (2) the qualitytest2, (3) the aspect ratio, and (4) the orthogonality. Qualitytest1 and qualitytest2 are the measurements of the volume over the edge length and skew, see Eqs. (1) and (2), respectively.

(1) $$B=V/L^{3}$$

where *B* represents the volume over the edge length, *V* represents tetrahedron volume and *L* represents shortest edge length.

(2) $$T_{skew}=\left(V_{ideal}-V\right)/V_{ideal}$$

where *T_skew_* represents the skew, *V* represents tetrahedron volume and *V_ideal_* is the volume according to the radius of the circumscribed ball.

The aspect ratio of tetrahedral mesh is the ratio of the longest edge length to the shortest edge length in Eq. (3).

(3) $$A_{ratio}=L_{longest}/L_{shortest}$$

where *A_ratio_* represents the aspect ratio, *L_longest_* represents longest edge length and *L_shortest_* represents shortest edge length.

**Fig. 4:**
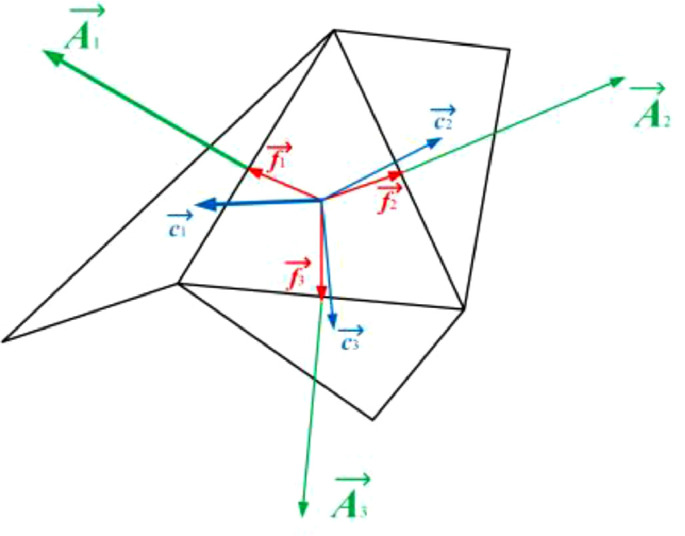
Vectors used to compute the ortho skew quality of a tetrahedron.

The ortho skew quality for meshes is computed using the face normal vector, and the vector from the tetrahedron centroid to each of the faces. Fig. 4 illustrates the vectors used to determine the ortho skew quality for a mesh.

The ortho skew for a tetrahedron is computed as the maximum of the following quantities computed for each face *i* in Eq. (4).

(4) $$O_{ratio}=\frac{\;\underset{A_{i}}{\to }.\underset{F_{i}}{\to }\;}{\underset{|A_{i}|}{\to }.\underset{|F_{i}|}{\to }}$$

where $\vec{A_{i}}$ is the face normal vector and $\vec{F_{i}}$ is a vector from the centroid of the tetrahedron to the centroid of that face.

## Result

### Application Case 1

This model is enclosed by 21 triangulated surfaces and contains eight strata. There are two thin layers and a fault in this model. For this kind of geological model, different mesh sizes for different subdomains based on the user's needs can generally better fulfil the engineering requirements. We provide different mesh size parameters. After the meshing process, we obtain a series of tetrahedral meshes. We then import these meshes into FLAC^3D^.

**Fig. 5:**
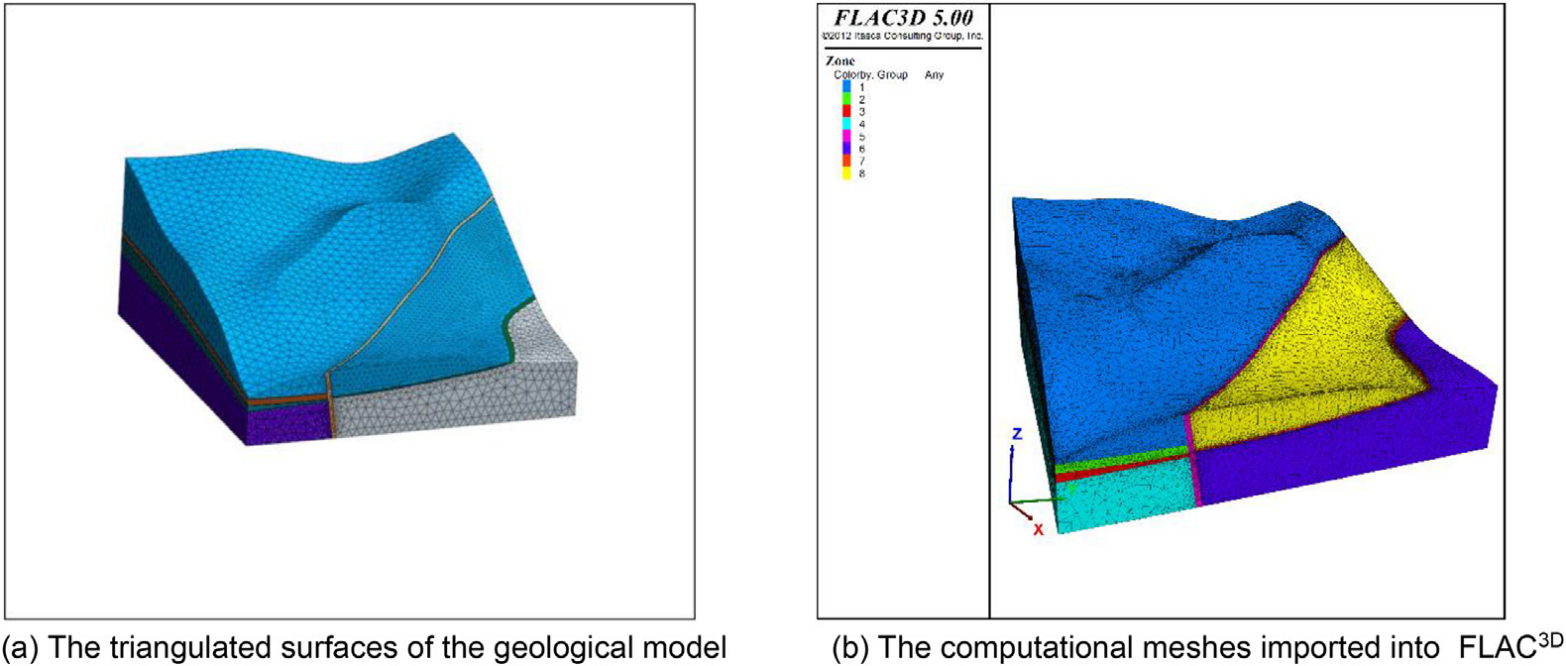
The input geological model and generated computational mesh in application case 1.

**Fig. 6:**
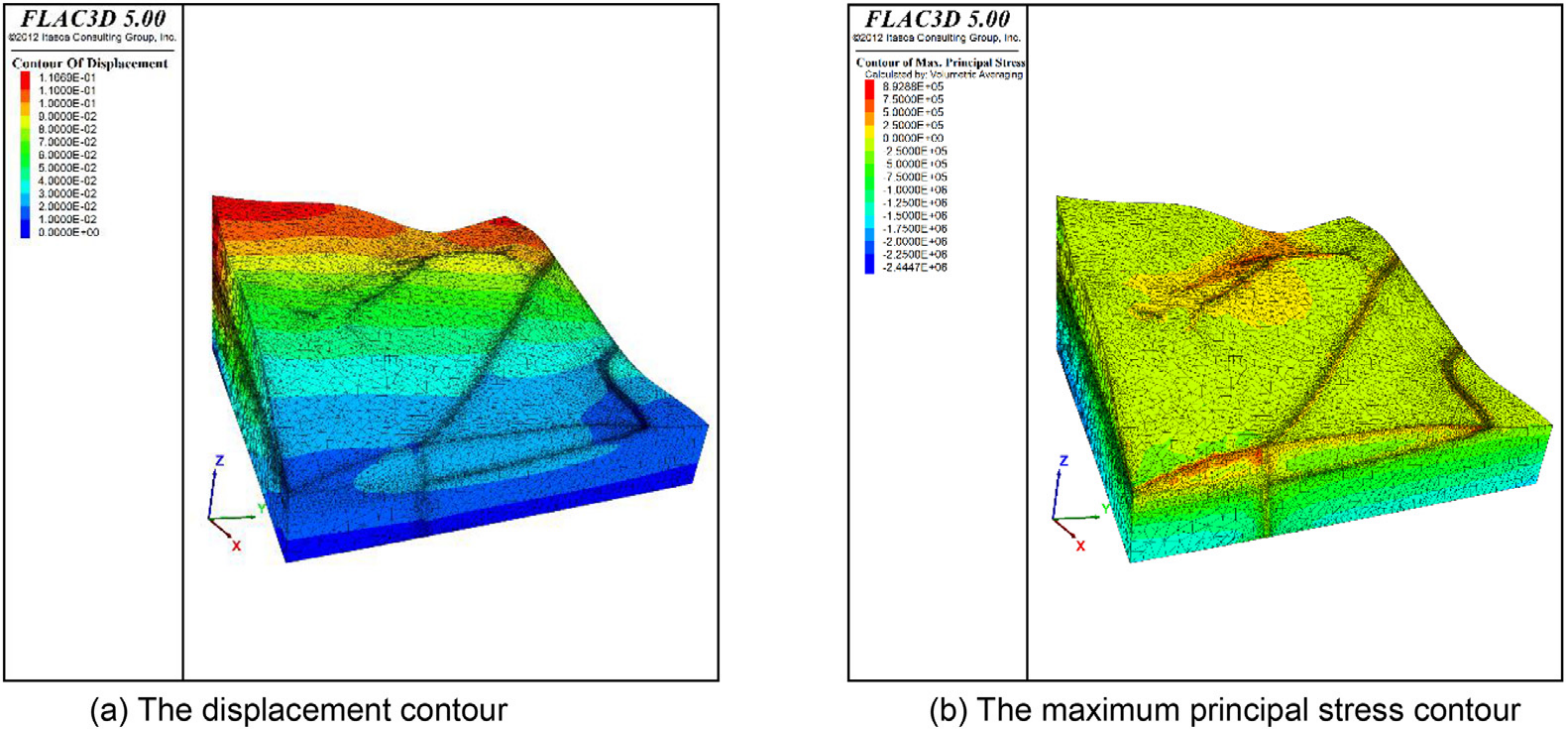
Numerically calculated results in application case 1.

**Fig. 7:**
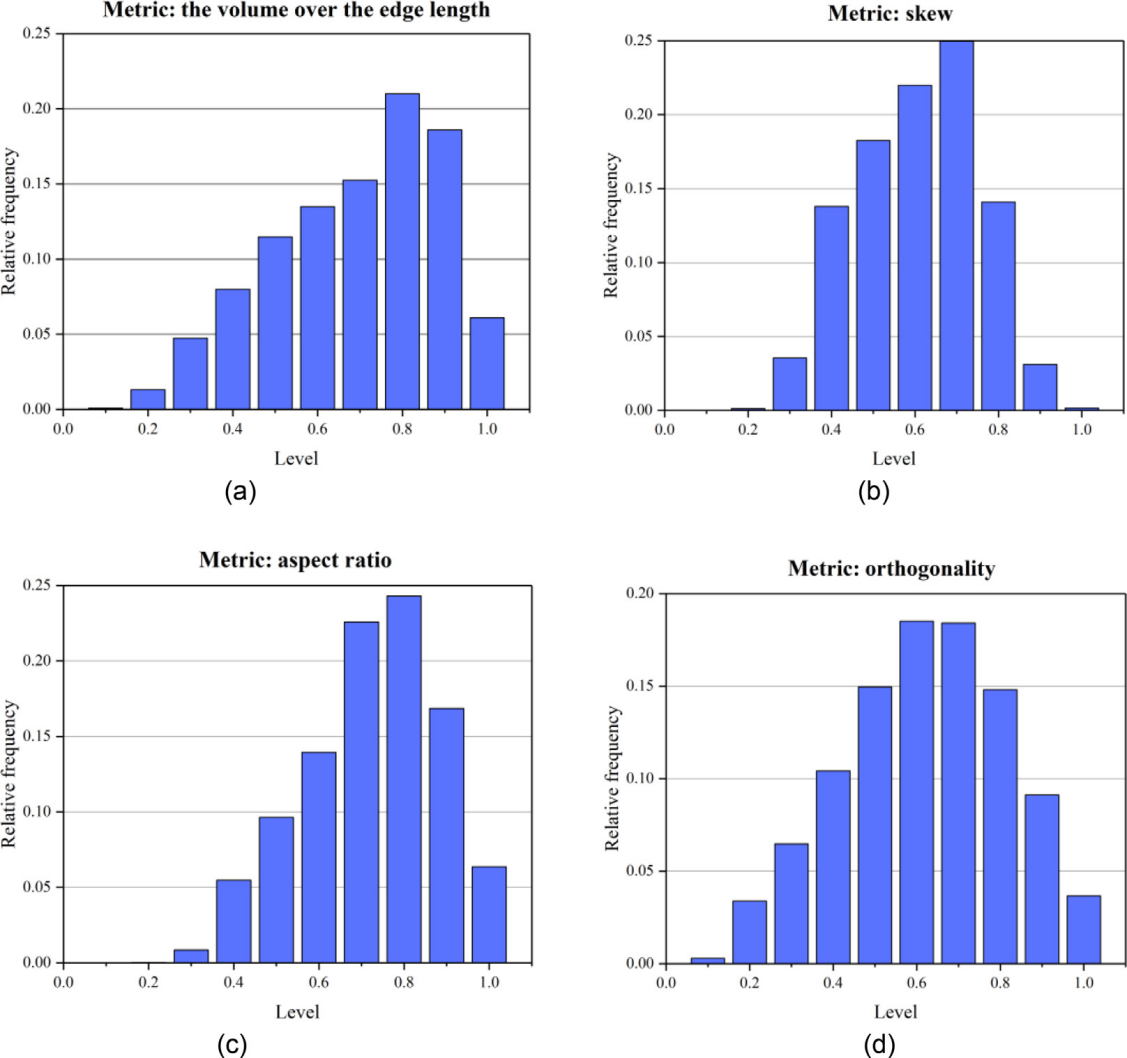
Statistics of the quality of computational mesh in application case 1.

Fig. 5 shows the mesh generated by the presented method. The mesh size for thin layers and faults is set to 4 and the size of the meshes of other strata is set to 10. Finally, the model is divided into 62,994 nodes, and 312,542 tetrahedral meshes. The boundary condition is the same as that in the above application cases. The Mohr-Coulomb model was used to simulate stress and strain under a self-weight state. The maximum displacement is approximately 0.1 m (Fig. 6). A mesh quality analysis based on the value distribution of the meshes is shown in Fig. 13. Approximately 68% of all the meshes have values in the range from 0.5 to 1.0 and the proportion of values less than 0.2 is extremely low in Fig. 7(a). The distribution of mesh quality generally has a peak at the value at 0.6 in Fig. 7(b) and the proportion of meshes with values greater than 0.5 is over 65% in Fig. 7(c). Smooth distributions are also visible in Fig. 7(d). In general, the distribution of mesh quality generally has a peak at a value from 0.6 to 0.8. The majority of meshes have a quality value between 0.5 and 1.0. More than 60% of the meshes have a value greater than 0.4. Thus, all the tetrahedral meshes are able to satisfy the requirements of numerical simulations. The results also show that these computational meshes can be used for numerical simulation in other kinds of commercial software.

### Application Case 2

**Fig. 8:**
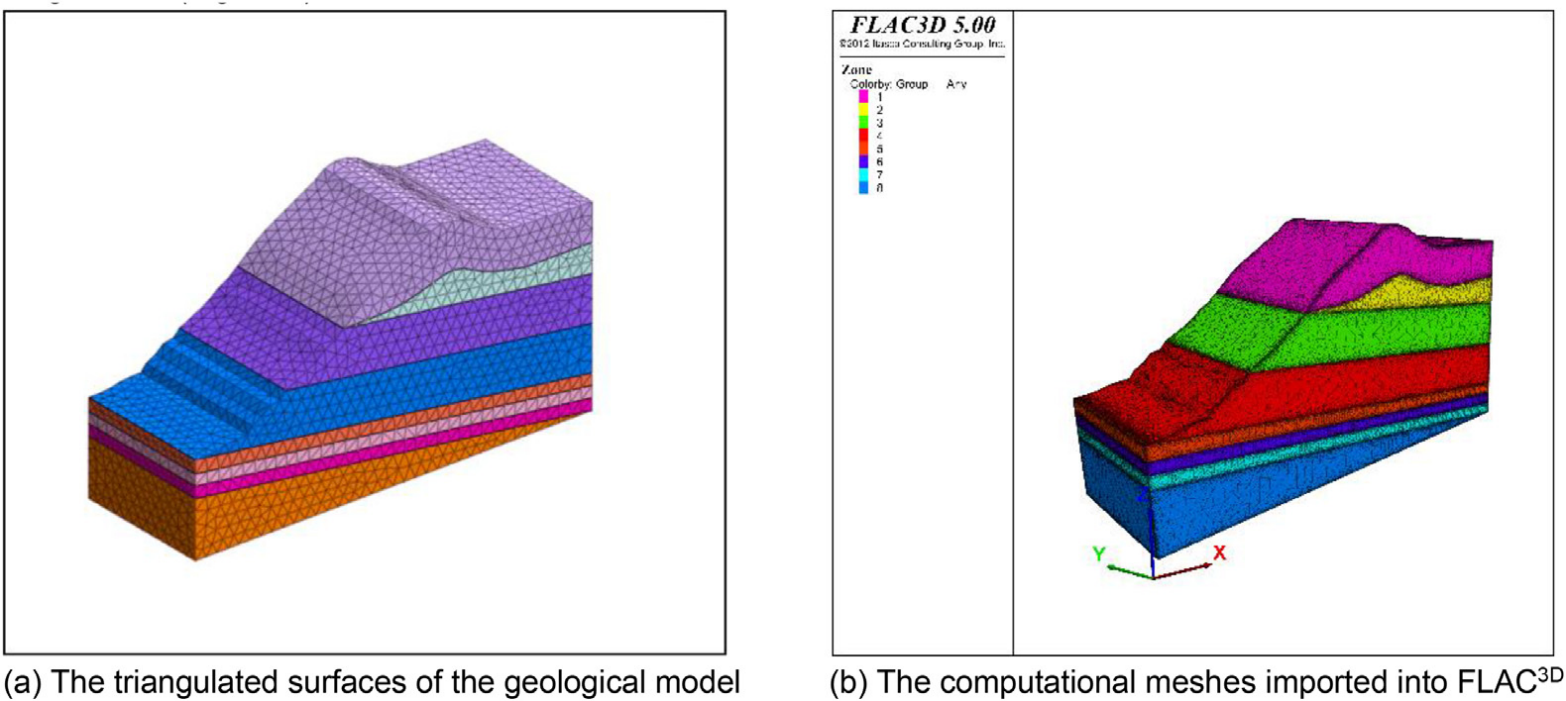
The input geological model and generated computational mesh in application case 2.

**Fig. 9:**
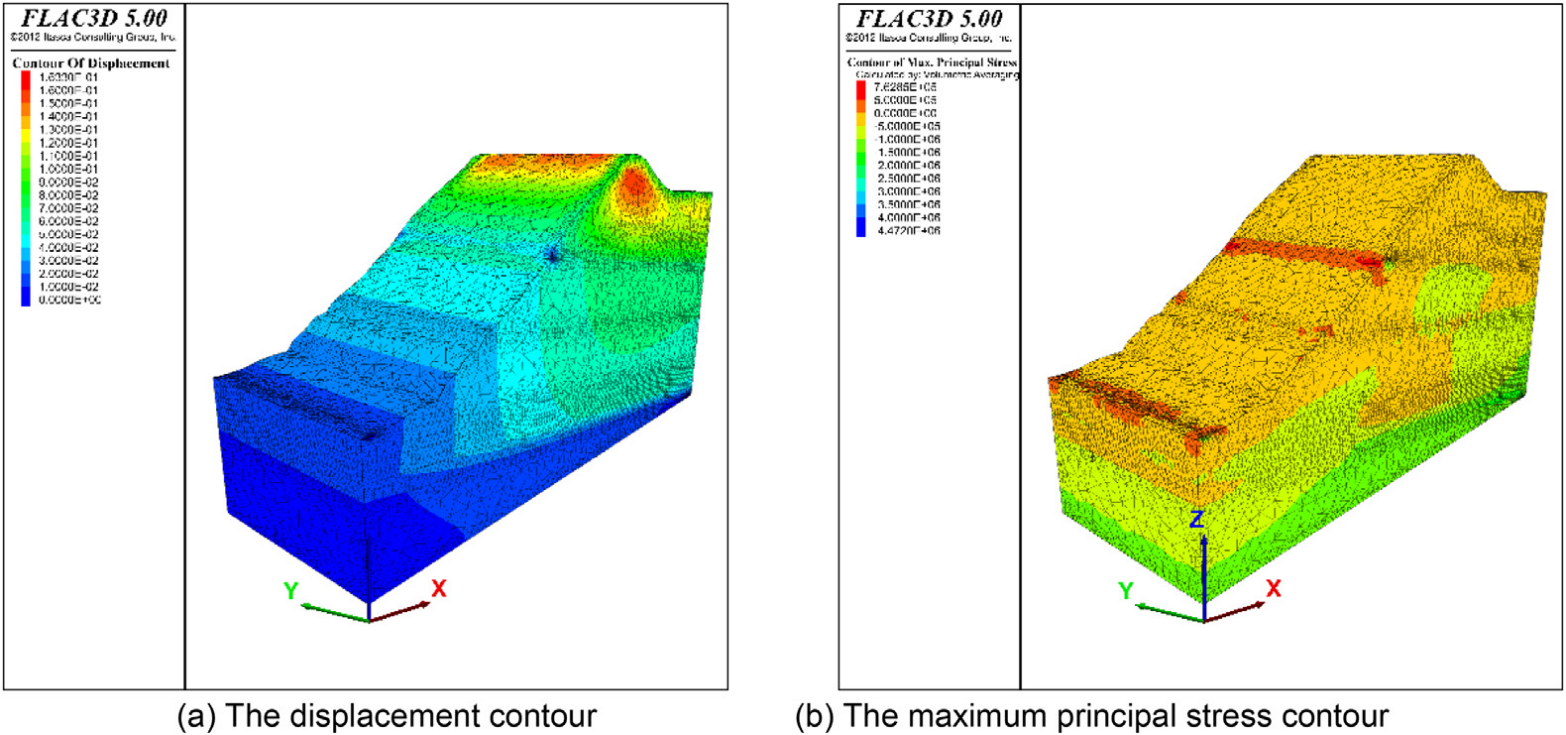
Numerically calculated results of application case 2.

The geological model represents a slope located in Shanxi Province in China. The computational mesh comprises 54496 vertices, 70970 triangles, and 259652 tetrahedral meshes (Fig. 8). The X-axis side and Y-axis side were adopted to constrain the X-direction and Y-direction displacement respectively. The bottom boundary of the model fixed the *Z*-direction displacement, and the top boundary is the free boundary. The computational results show the displacement contour and the maximum principal stress contour under the self-weight state (Fig. 9).

**Fig. 10:**
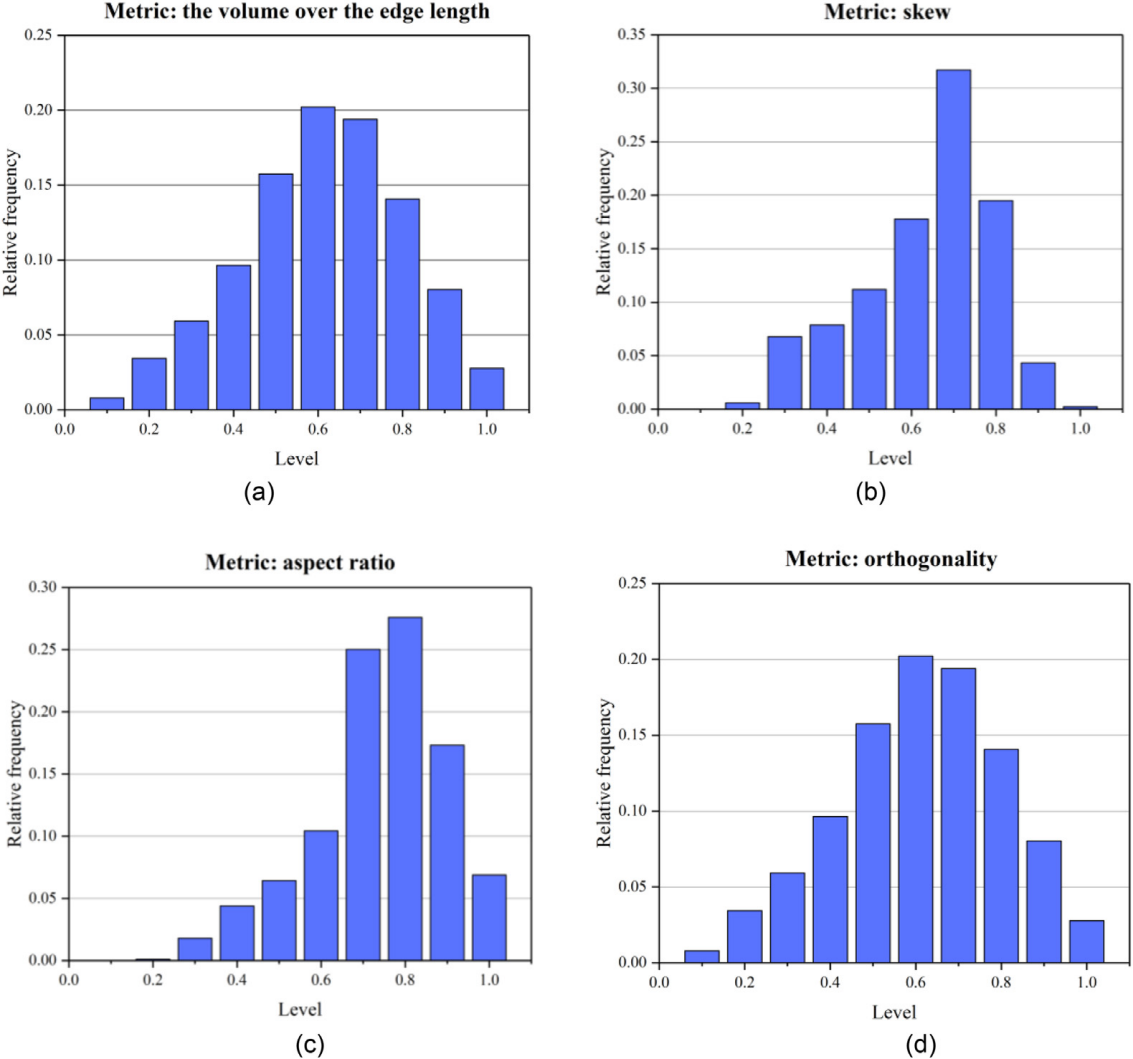
Statistics of the quality of computational mesh in application case 2.

**Fig. 11:**
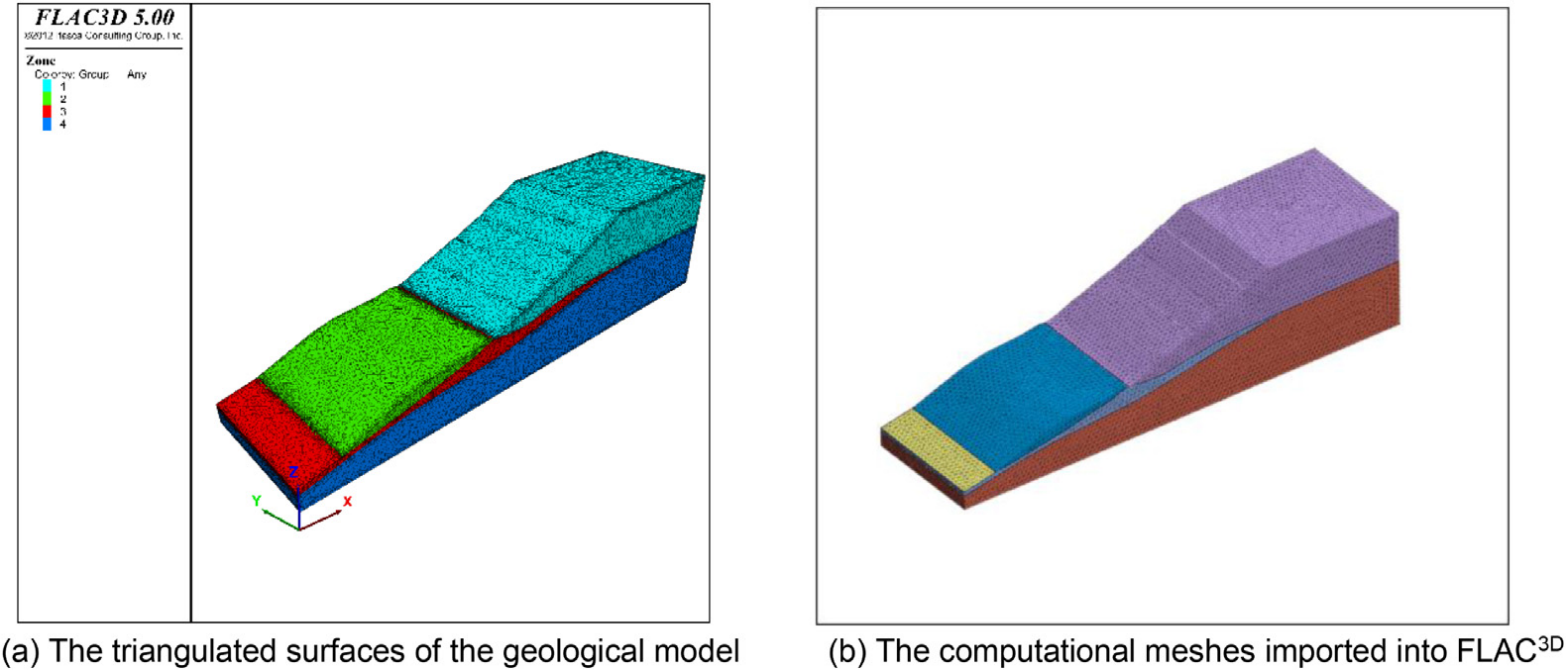
The input geological model and generated computational mesh in application case 3.

Mesh quality analysis is performed based on the value distribution of the meshes in Fig. 10. In Fig. 10 (a) and 10 (d), the distribution of the mesh quality is smooth. Approximately 65% of all the tetrahedral meshes have values in the range from 0.5 to 1.0. Less than 5% of the mesh has a value less than 0.2. In Fig. 10 (b) and 10 (c), the distribution still has a peak at a value between 0.6 and 0.8, which indicates a lower proportion of distorted meshes. Therefore, all the computational tetrahedral meshes are able to satisfy the requirements of numerical simulations (Fig. 11).

### Application Case 3

**Fig. 12:**
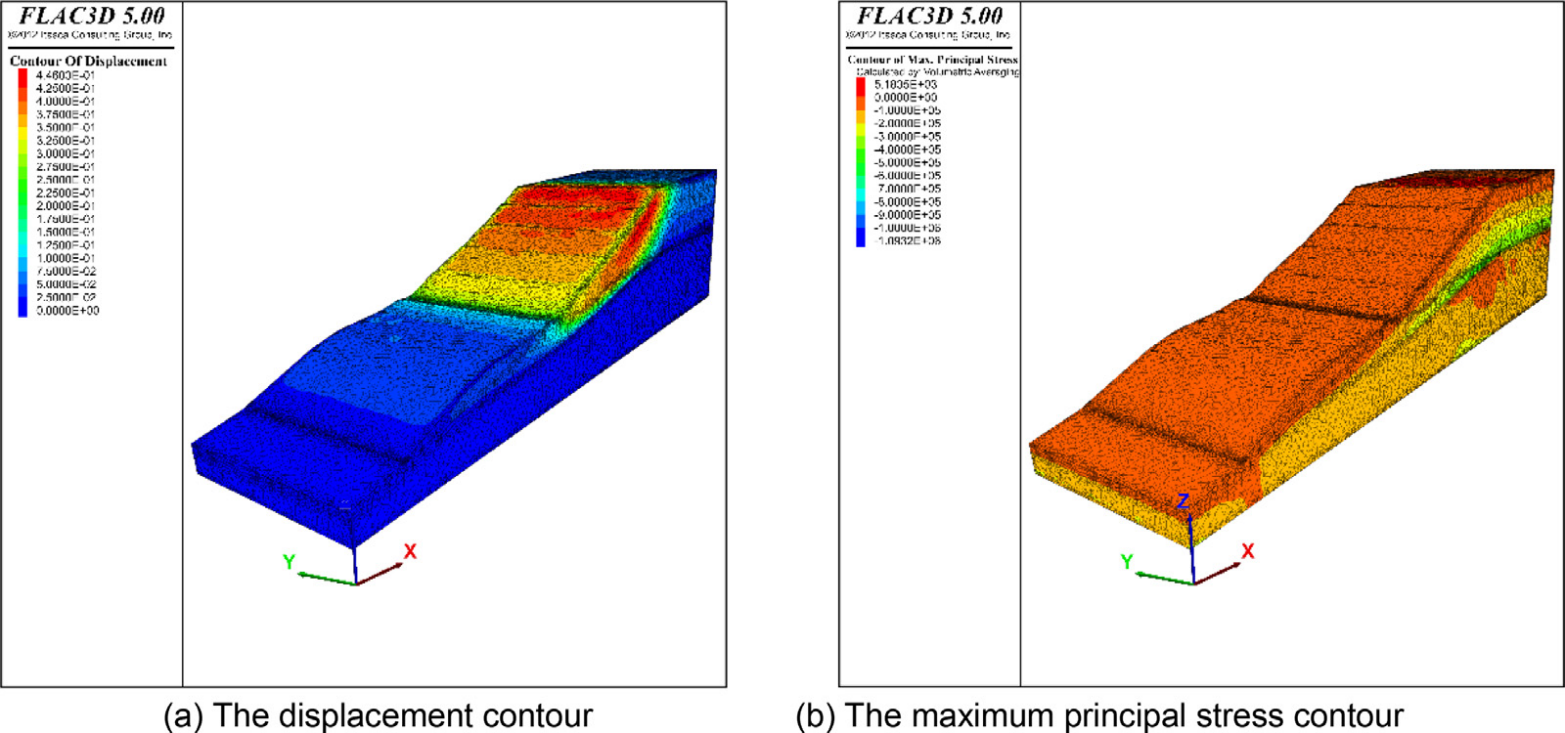
Numerically calculated results of application case 3.

**Fig. 13:**
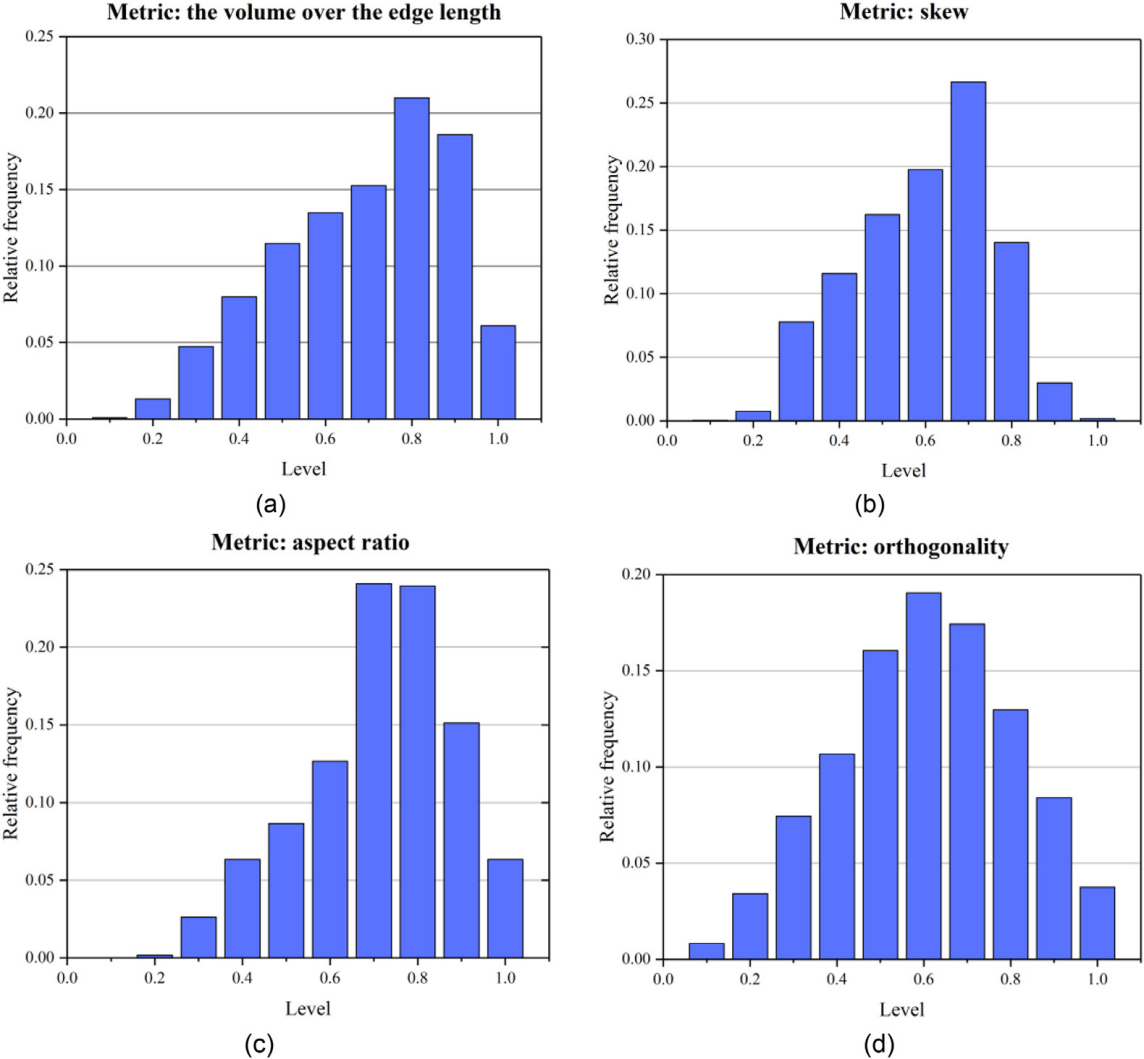
Statistics of the quality of computational mesh in application case 3.

This slope contains a large deformation, developing cracks and bulging, and is taken as a typical example for stability analysis. The computational meshes comprise 99338 vertices, 203098 triangles, and 365631 tetrahedral meshes. The boundary condition is the same as that in application case 1. After the numerical simulation, the displacement and the maximum principal stress contour under the self-weight state are shown in Fig. 12. The maximum displacement is approximately 0.7 m. Fig. 13 provides the quality distribution of the tetrahedral meshes. A mesh quality analysis based on the value distribution of the meshes is shown in Fig. 13. Approximately 55% of all the meshes have values in the range from 0.5 to 1.0 and the proportion of values less than 0.1 is extremely low (Fig. 13(a)). The distribution of mesh quality has a peak at a value between 0.6 and 0.8 and the proportion of meshes with a good aspect ratio is over 60% in Fig. 13(c). In general, the majority of meshes had a high-quality value between 0.5 and 1.0, and smooth distributions are also visible in Fig. 13, which indicates the high quality in the model. Thus, all the tetrahedral meshes are able to satisfy the requirements of numerical simulations.

## Discussion

### Effectiveness of the presented method

Mesh generation of a geological model can be achieved by the presented method. Currently, this method can generate high-quality tetrahedral meshes of geological models consisting of triangulated surfaces. These tetrahedral meshes meet the mesh criteria by as much as possible. The output meshes are reasonably well represented by geological features.

Examples demonstrate that the method is practical. These meshes can be used in other simulation software. We import these computational meshes into FLAC^3D^ and set boundary conditions for the model. The simulation results are obtained from FLAC3D which is often used for numerical simulation; and the results of the calculation show that these tetrahedral meshes of the geological model can be used in stability analysis of rock and soil masses. Thus, the simulation results are also reliable.

Measuring the mesh quality is important for this method. For the different mesh generation methods, the measurement metrics of the element quality are similar, which usually include the aspect ratio and the orthogonality. Besides, we also add the metrics of the volume over the edge length and skew. These quantities are mainly used to determine the quality based on the degree and edge length of elements and to identify degenerate elements. In general, the majority of meshes had a high-quality value and smooth distributions are also visible from the statistic of mesh quality metrics, which indicates the high quality in the model.

### Advantages and shortcomings

The method of utilizing CGAL is easy to reproduce. Moreover, compared with other methods for mesh generation, this method focuses only on mesh generation for geological models. Thus, it is easy to implement.

There is a procedure of optimizing mesh in the process of mesh generation for the improvement of the average quality of the mesh. The method can eliminate some distortion meshes effectively and can improve the average quality of the mesh after optimization. There are four inherent optimization functions in CGAL. The method can call its optimization functions to improve the overall mesh quality in this process. The functions not only focus on remove some badly shaped local tetrahedral meshes but also attempt to improve the overall mesh quality. In this process, some boundary edges typically need to be preserved in the input domain. The optimization process is automatically executed and the final output is optimized meshes.

Tetrahedral meshes with different mesh sizes can be generated in different subdomains, but there is also a shortcoming in these tetrahedral meshes. In each subdomain, adaptive computational meshes cannot be generated. That is the mesh size is almost the same, which increases the number of triangular and mesh vertices. Such largescale tetrahedral meshes will lead to an increased calculation time and a decreased computational efficiency of the model.

### Outlook and Future Work

In the future, we plan to propose a much more adaptive mesh generation method for very complex geological models. The mesh generation time will also be significantly reduced with that method. According to the features of the geometric model, quite adaptive high-quality tetrahedral mesh generation and smoothing [5,6] of the geological model will be implemented.

## Conclusion

We have presented a method for generating high-quality tetrahedral meshes of geological models to be used in the stability analysis of rock and soil masses. The input is a geological model consisting of triangulated surfaces, and the output is a high-quality tetrahedral mesh of the geological model by utilizing CGAL. To demonstrate the effectiveness of the method, we implement it to generate a series of computational meshes in geological model, and we analysed the stabilities of the rock and soil slopes on the basis of the generated tetrahedral mesh models. The applications demonstrated the effectiveness and practicability of the presented method.

**Additional information:**

## Introduction

Numerical simulation has become an indispensable means to solve scientific problems and conduct scientific research in many fields. High-quality meshes are key to the success of all numerical simulations [7,8]. High-quality meshes are usually a balance between quality and calculation time, and low-quality, extremely thin or distorted elements often prevent the convergence of numerical calculations and increase analytical errors. Among the many automatic mesh generation algorithms, Delaunay triangulation [9,10] has been widely developed and applied due to the high quality of the generated mesh and easy implementation of the algorithm.

Various commercial software programs have been developed and applied to numeral calculations in recent years [11], [12], [13], [14]. For example, HyperMesh can generate all the mesh types required in finite element calculations and ANSA is similar to HyperMesh. It is widely used in the automotive field. The advantage of these programs lies in the generation of surface meshes. ANSYS ICEM CFD is a full-featured CFD mesh generation tool. It supports not only hexahedral meshes but also tetrahedral meshes, pentagonal meshes, and triangular prisms, which are sufficient for mesh generation of complex geometric model. It is more focused on generating fluid meshes. GridPro is suitable for geometric models from which structural meshes are easy to generate, but the software cannot generate unstructured meshes. Pointwise produces extremely high-quality structured and unstructured meshes.

Several open source mesh generators have also been developed, such as CGAL, TetGen, Ani3D, MFEM, NETGEN, and Stellar. These packages are widely used in various areas that require geometric computation. TetGen is very popular for generating three-dimensional tetrahedral meshes and Delaunay triangulations [15]. Ani3D can also generate unstructured tetrahedral meshes and can be used to generate quasi-optimal meshes [16]. MFEM was designed for finite element methods [17]. NETGEN generated meshes based on abstract rules [18]. Stellar is often used to optimize tetrahedral meshes to produce high quality tetrahedral meshes [19].

In the field of geotechnical engineering, geological models can be divided into computational tetrahedral, hexahedral or hybrid meshes [20,21]. Much work has focused on hexahedron-dominant mesh generation methods [22]. Meng [23] presented a novel link-Delaunay-triangulation method to achieve geometric and topological consistency. Indirect methods [24,25] were proposed to convert Delaunay triangular meshes into quadrilateral meshes by combining adjacent pairs of triangles. Constrained triangulated surfaces can be used to mesh a three-dimensional geological model by using a series of tetrahedral meshes [26], [27], [28].

Commercial software can generate high-quality meshes, but these programs are expensive. Compared with the above open source packages, CGAL is based on Delaunay refinement. Thus, there are remeshed surfaces in the model. CGAL also provides two global optimistic functions and two local optimistic functions to improve the average quality of the mesh. Therefore, method for mesh generation based on CGAL is easily implement.

## Background: the CGAL library

The Computational Geometry Algorithms Library (CGAL) [9] is an open source C++ graphics algorithm library that provides underlying half-edge data structures. It is a very convenient and efficient program for iteratively traversing points, edges and faces. CGAL provides data structures and algorithms related to computational geometry, such as Voronoi diagrams, triangulations, Boolean operations on polygons and polyhedrons, curve finishing and its applications, mesh generation, geometry processing, and shape analysis.

Because of its open source and portable characteristics, it has already been widely used in computer-aided design and modelling, computer graphics, geographic information systems, numerical methods, medical imaging, and other fields that require geometric computation.

CGAL mainly comprises three components: kernels, computational geometry basic data structures and algorithms and non-geometric tools. CGAL is a header-only C++ template library and is based on a generic programming paradigm of concepts and models. Each of the main functions in CGAL exists as a class template. The input and output functions in each class are overloaded. Therefore, different data types can be easily and flexibly handled with overloaded functions.

The presented method utilizing CGAL, is committed to the generation of isotropic simplified meshes for discretized 3D domains constructed by a series of triangulated surfaces. The 3D space to be meshed is called the domain, and it is required that it be bounded and defined by a collection of initial triangulated surfaces. The domain may be subdivided into several subdomains. Some points and edges may need to be approximated in the mesh; these points and edges are defined as indispensable 1-dimensional features and 0-dimensional features respectively. In addition, 3-dimensional features and 2-dimensional features are defined as subdomains and boundary triangulated surfaces, respectively.

## Declaration of Competing Interest

The Authors confirm that there are no conflicts of interest.

## References

[1] Chen L. (2004). Mesh Smoothing Schemes Based on Optimal Delaunay Triangulations. International Meshing Roundtable.

[2] Du Q., Faber V., Gunzburger M. (1999). Centroidal Voronoi tessellations: Applications and algorithms. Siam. Rev..

[3] Alliez P., Cohensteiner D., Yvinec M., Desbrun M. (2005). Variational tetrahedral meshing. International Conference on Computer Graphics and Interactive Techniques.

[4] Li T., Li S., Qiu X., Chen W. (2004). Application of fast lagrangian analysis of continua to researching on safe rock covers of Xiamen subsea tunnel. Rock and Soil Mechanics.

[5] Mei G., Cuomo S., Tian H., Xu N.X., Peng L.J. (2018). MeshCleaner: A Generic and Straightforward Algorithm for Cleaning Finite Element Meshes. Int. J. Parallel Program..

[6] K. Zhao, On the Accelerating of Two-dimensional Smart Laplacian Smoothing on the GPU, J. Inf. Comput. Sci. 12 (2015), 5133-5143, doi:10.12733/jics20106587.

[7] Deng Z., Wang Y., Zhao G., Zhang J. (2012). Unstructured Surface Mesh Generation for Topography Using Interpolating Surface Modeling. Adv. Future Comput. Control Syst..

[8] Vasilevskii Y.V., Lipnikov K.N. (1999). An adaptive algorithm for quasioptimal mesh generation. Comput. Math. Math. Phys..

[9] Alliez P., Fabri A., Fogel E. (2009). Computational geometry algorithms library. ACM SIGGRAPH ASIA 2009 Courses.

[10] Bowyer A. (1981). Computing Dirichlet tessellations. Comput. J..

[11] Guo J.W., Ding F., Jia X.H., Yan D.M. (2019). Automatic and high-quality surface mesh generation for CAD models. Computer Aided Design.

[12] Zhang Y.X., Jia Y.F., Wang S.S.Y., Altinakar M. (2013). Composite Structured Mesh Generation with Automatic Domain Decomposition in Complex Geometries. Eng. Appl. Comput. Fluid Mech..

[13] Vasilev E., Lachinov D., Grishin A., Turlapov V. (2018). Fast tetrahedral mesh generation and segmentation of an atlas-based heart model using a periodic uniform grid. Russ. J. Numer. Anal. Math. Model..

[14] Meng X., Zhou K., Li J., Yang Q. (2013). Triangular framework mesh generation of 3D geological structure. Int. Conf. Machine Vis..

[15] Si H. (2015). TetGen, a Delaunay-Based Quality Tetrahedral Mesh Generator. ACM Trans. Math. Softw..

[16] Yoon C. (2019). An Automated Adaptive Finite Element Mesh Generation for Dynamics. Journal of Earthquake Engineering Society of Korea.

[17] Vassilevski P.S. (2002). Sparse matrix element topology with application to AMG(e) and preconditioning. Numer. Linear Algebra Appl..

[18] Schöberl J. (1997). NETGEN An advancing front 2D/3D-mesh generator based on abstract rules. Comput. Vis. Sci..

[19] Klingner B.M., Shewchuk J.R. (2007). Aggressive Tetrahedral Mesh Improvement. Proceedings of the 16th international meshing roundtable.

[20] Hammond G.E., Lichtner P.C., Mills R.T. (2014). Evaluating the performance of parallel subsurface simulators: An illustrative example with PFLOTRAN. Water Resour. Res..

[21] Jansen G., Sohrabi R., Miller S.A. (2017). HULK – Simple and fast generation of structured hexahedral meshes for improved subsurface simulations. Comput. Geosci..

[22] Pei X.B., Xu N.X. (2014). Hexahedron-dominant Mesh Generation for Blocks with Constrained Triangulated Boundary Surfaces. Appl. Mech. Mater..

[23] Sarrate J., Ruiz-Gironés E., Roca X. (2014). Unstructured and Semi-Structured Hexahedral Mesh Generation Methods. Comput. Technol. Rev..

[24] Sun L., Yeh G.T., Lin F.P., Zhao G.Q. (2015). Automatic quadrilateral mesh generation and quality improvement techniques for an improved combination method. Comput. Geosci..

[25] Araujo C., Celes W. (2014). Quadrilateral Mesh Generation with Deferred Constraint Insertion. 23rd International Meshing Roundtable.

[26] Miranda A.C.D., Lira W.W.M., Marques R.C., Pereira A.M.B., Cavalcante-Neto J.B., Martha L.F. (2015). Finite element mesh generation for subsurface simulation models. Eng. Comput..

[27] Eller D., Tomac M. (2016). Implementation and evaluation of automated tetrahedral prismatic mesh generation software. Comput. Aided Des..

[28] Xing H.L., Liu Y. (2014). Mesh Generation for 3D Geological Reservoirs with Arbitrary Stratigraphic Surface Constraints. 2014 International Conference on Computational Science.

